# A qualitative analysis of unintended effects of a digital conditional cash transfer intervention to encourage healthcare utilization in Southern Madagascar

**DOI:** 10.1186/s12913-025-12354-z

**Published:** 2025-02-05

**Authors:** Mara Anna Franke, Anne Neumann, Kim Nordmann, Daniela Suleymanova, Onja Gabrielle Ravololohanitra, Samuel Knauss, Julius Valentin Emmrich

**Affiliations:** 1 Global Digital Health Lab at Charité Center for Global Health, Charité – Universitätsmedizin Berlin, Berlin, 10117 Germany; 2 Ärzte Für Madagaskar E.V, Leipzig, Germany; 3 Rheinisch-Westfälische Technische Hochschule Aachen, Aachen, Germany; 4 Paris Institute of Political Studies, Paris, France; 5 Doctors for Madagascar, Antananarivo, Madagascar; 6 Heidelberg Institute of Global Health, Heidelberg University, Heidelberg, Germany; 7 Berlin Institute of Health at Charité - Universitätsmedizin Berlin, Berlin, Germany

**Keywords:** Sub-Saharan Africa, Conditional cash transfers, Healthcare utilisation, Global health

## Abstract

**Introduction:**

Cash transfer interventions, including those using mobile money, are becoming increasingly widespread, particularly in Sub-Saharan Africa. As such interventions can have significant positive and negative unintended consequences, further analyses are needed to identify these consequences.

**Methods:**

We investigated the unintended consequences of a digital conditional cash transfer intervention implemented at fifteen health facilities in Southern Madagascar. The intervention offered partial cost coverage for patients seeking care for potentially life-threatening conditions, accidents and injuries, maternal or pediatric care between February 2021 and June 2022. We conducted a qualitative analysis of in-depth interviews with policymakers, healthcare providers, (non-) beneficiaries of the intervention, and staff that implemented the intervention using reflexive thematic analysis.

**Results:**

We identified three key positive and three key negative unintended consequences of the intervention. The key positive unintended consequences were: i) improved quality of care, ii) improved interpersonal relationships, including between patients and providers and between healthcare providers, and iii) digital skills development of healthcare providers and increased trust in mobile money. The three key negative consequences we identified were i) facility overcrowding, ii) an increase in costs of care, and iii) cases of hospital imprisonment.

**Conclusions:**

Designers and implementers of future (digital) cash transfer interventions should carefully consider and proactively seek to leverage the positive and mitigate the negative unintended consequences of cash transfer interventions for healthcare such as those highlighted in our work.

**Supplementary Information:**

The online version contains supplementary material available at 10.1186/s12913-025-12354-z.

## Background

Madagascar, an island nation with 29 million inhabitants is one of the least developed countries globally, it currently ranks 173rd out of 190 countries on the human development index [1]. Extreme poverty is rife; more than 80% of the population lives below the international poverty line of USD 2.15 a day (2017 purchasing power parity (PPP)) [2]. Only approximately 2% of the population is covered by a health insurance scheme and more than 40% of all healthcare costs in Madagascar are paid out-of-pocket (OOP) surpassing the Sub-Saharan African (SSA) regional average [3, 4]. Financial constraints are the primary reason for foregoing necessary medical care, with 58% of individuals from the highest wealth quintile and 78% from the lowest citing financial barriers as the main obstacle in accessing healthcare [5]. Infectious diseases, including malaria, respiratory diseases, and diarrheal diseases are a leading cause of mortality in the country [4]. Tuberculosis is highly prevalent with 233 cases per 100,000 population [2] and the maternal and under 5 mortality rate (392 per 100,000 and 66 per 1,00 live births respectively) remain high [2].

The Covid-19 pandemic put additional strain on the Malagasy economy, reducing foreign trade and causing a 4.2% contraction in gross-domestic product (GDP) [6]. Consequently, the prevalence and depth of poverty in the country increased even further and unemployment rates rose [6]. The effect on healthcare utilization in the country was equally severe, with a significant reduction in the use of key healthcare services, particularly for outpatient services, and reported delays in healthcare seeking, leading to severe complications, such as intestinal perforations from typhoid fever in the capital city of Antananarivo [7, 8]. Given the already high financial barriers to care pre-pandemic, it is likely that the economic strain caused by the pandemic further aggravated financial barriers to care, even though empirical evidence is lacking.

In this context, a German-Malagasy non-governmental organization (NGO) implemented a digital, conditional cash-transfer intervention to reduce financial barriers to care for patients if they accessed care for a potentially life-threatening health condition, accidents and injuries, maternity or pediatric care at a participating health facility during the intervention period. The intervention is explained in detail in the methods section, but briefly, eligible patients received a partial (80%) cost coverage for drugs and medical consumables.

The number of cash transfer interventions in international development is rising, including conditional cash transfers for healthcare seeking and the use of digital technologies such as widespread mobile money services. Multi-faceted analyses of such interventions, including their unintended consequences, are therefore becoming increasingly important. Unintended consequences can be understood as positive or negative consequences that were not foreseen, planned, or desired by the original designers and implementers of an intervention [9]. Despite not being originally envisioned or planned, unintended consequences can have significant influences on target populations and their health and well-being, sometimes more severely than the intended consequences of an intervention [10]. While there is increasing evidence examining the effectiveness of cash transfer programs for health [11–15], the evidence on their unintended effects is limited. Previous studies have, however, some key unintended consequences of cash transfers targeted at improving child health, where children were kept underweight on purpose to continue the family benefiting from the cash transfer program [16]. Similarly, evidence from Malawi highlights a cash transfer program increasing, risky sexual behaviour among male beneficiaries [13].

Similar evidence exists from cash transfer programs targeting other areas of intervention, for example, violence reduction in Latin America [17] and entrepreneurship in low-income settings [18]. For example, there was increased instead of decreased violence in the first program, and there were both positive spillover effects and negative unintended effects caused by envy among community members in the latter [17, 18].

In this paper, we thus aim to analyze the unintended consequences of a digital conditional cash transfer intervention for healthcare services in Southern Madagascar during the COVID-19 pandemic.

## Methods

### Study area and context

The Malagasy healthcare system is organized in 4 tiers: Community health workers (CHWs) provide health education and basic care at the community level. Private and public basic health centers (CSBs, centers de santé de base) provide basic outpatient, maternal, and pediatric care. District referral hospitals provide secondary care, including surgical care. More specialized care is only available at regional and national referral hospitals [19]. Private and faith-based healthcare providers play an essential role in healthcare provision, especially at the secondary care level in Madagascar. Over 35% of contacts with healthcare services happen in the private sector, including faith-based facilities [3], particularly in remote and rural areas.

### Intervention description

To mitigate the adverse impacts of the COVID-19 pandemic on healthcare utilization and the financial stability of healthcare institutions, the NGO Doctors for Madagascar (DFM) introduced a digital conditional cash transfer intervention for healthcare named “Tosik’Aina” (meaning vital subsidy in English).

**Fig. 1:**
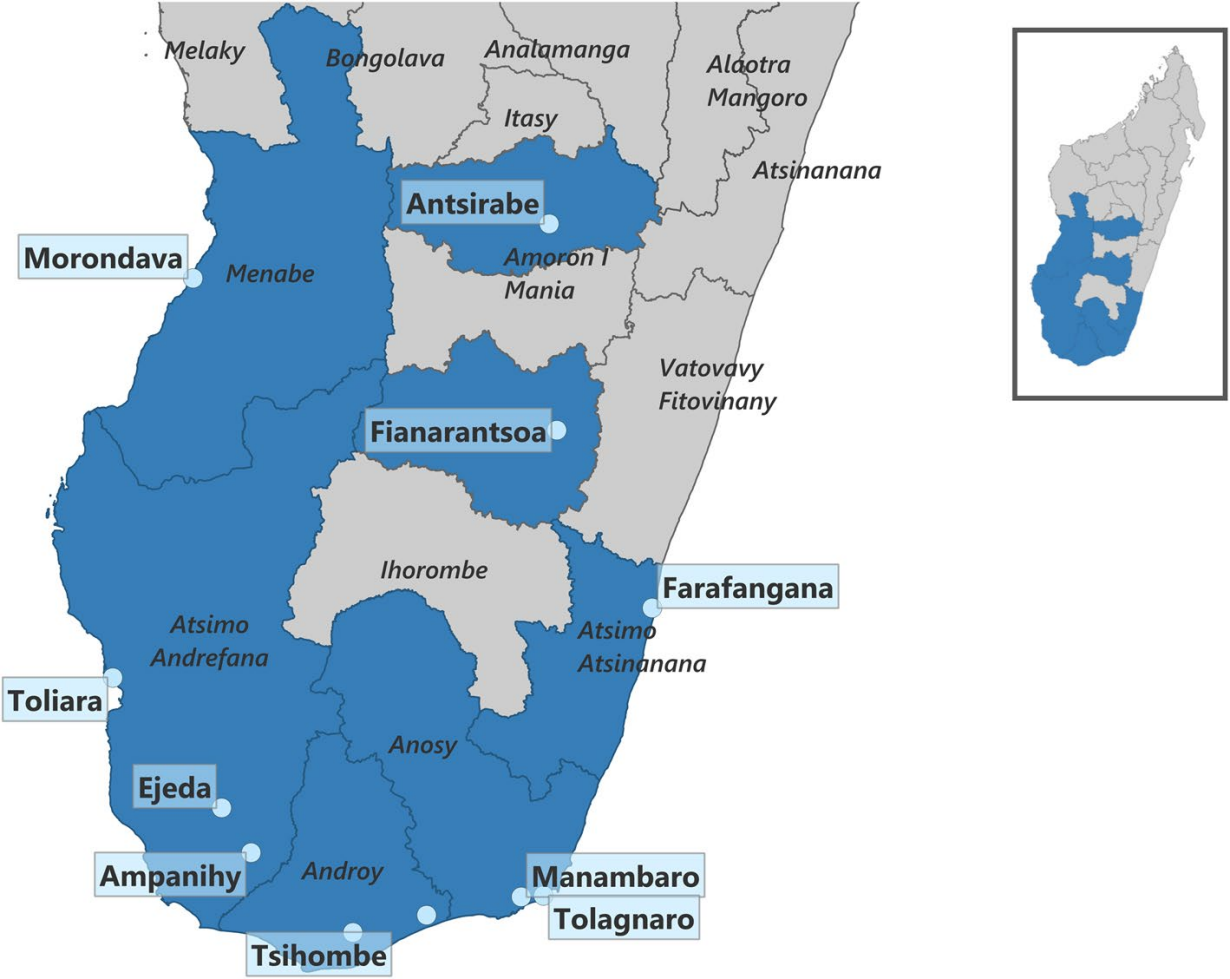
Map of participating facilities participating in Southern Madagascar. Map of municipalities where facilities participating in a digital conditional cash transfer intervention for enabling healthcare utilization in Southern Madagascar, February 2021-July 2022 are located. Regions with participating healthcare facilities marked in blue, regions without participating healthcare facilities marked in gray. Region names are in italics, municipality names are in bold. Municipalities with participating health centers are marked with light blue dots. The municipality of Tolagnaro held five participating health facilities. Inlet in the top right: Madagascar with study regions marked in blue

The intervention was implemented at 15 health centers located in 7 regions of Southern Madagascar. Facilities were accepted for the intervention based on their interest in participating, which they could register with the implementing NGO. Any type of facility (public, private, faith-based) or level of care provided (primary or secondary) could participate in the intervention. The inclusion of the facilities was based on their location (underserved and poorer regions were preferred) and a history of previous successful collaboration between the NGO and the facility. No facilities that applied to participate in the intervention were declined and facilities were admitted to the intervention on a rolling basis. Figure 1 below shows the location of each facility participating in the intervention.

Patients who were eligible to benefit from the digital conditional cash transfer intervention were children under the age of 5, pregnant women, and individuals seeking care for acutely life-threatening medical or surgical conditions. Patients seeking routine care for chronic non-communicable diseases were not eligible to participate, however, patients with acute exacerbations of chronic non-communicable diseases were eligible if the case was deemed potentially life-threatening. These target groups were chosen as they are particularly vulnerable in the context of Madagascar. Preventable deaths among children under 5 and pregnant women are common and healthcare-seeking behavior for children is especially impacted by financial constraints [3, 4]. Patients with potentially life-threatening conditions were deemed eligible as they are particularly likely to suffer financial hardship when seeking healthcare in Madagascar, especially if they require surgical care [2–4].

Enrollment decisions were made by each facility’s medical staff, most commonly the head doctor and/or nurse of the center. Centres were given a non-exhaustive list of conditions that were eligible for support under the category of “life-threatening conditions”, which included conditions such as malaria, tuberculosis, appendicitis, etc., to aid their decision making. In case of uncertainty regarding the eligibility of a patient, facility staff could directly contact the NGO’s staff via text, messenger or telephone.

For eligible patients, the NGO covered 80% of the expenses for medications and consumables. Patients paid for the remaining 20% of the costs for medication and consumables, as well as any additional expenses such as consultation fees, laboratory fees, or accommodation costs.

Before the intervention began, price lists were obtained from each participating facility, outlining the costs of all drugs and consumables. Costs for other services, such as diagnostic procedures or surgical interventions, were not detailed on the price lists. During the project’s implementation, all claims were systematically compared against these predefined price lists, ensuring that costs remained within the agreed parameters.

To facilitate the financial administration, all payments of the intervention employed the digital mTOMADY platform, the largest digital platform for healthcare payments in Madagascar.

Eligible patients were required to create an account on the mTOMADY platform for which a SIM (Subscriber Identity Module) card with a linked mobile money account was required. SIM cards and the costs of opening a mobile money account were not covered by the intervention. Patients could then transfer funds into their mobile money account and from there to their linked mTOMADY account.

Once a patient completed the treatment and was discharged from care, the healthcare facility filed a claim containing the patient’s medical history and details of the care received, on the mTOMADY platform, by filling out a digital questionnaire and submitting photographs of patient invoices and medical charts. Usually, an administrative clerk employed by the facility and a designated project staff member employed by the NGO were responsible for filing the claims for the intervention and communicating with the NGO’s medical claims team. This required them to communicate and collaborate closely with the medical team at the facilities to collect patients’ medical charts and reply to any clarification requests from the central claims team.

**Fig. 2:**
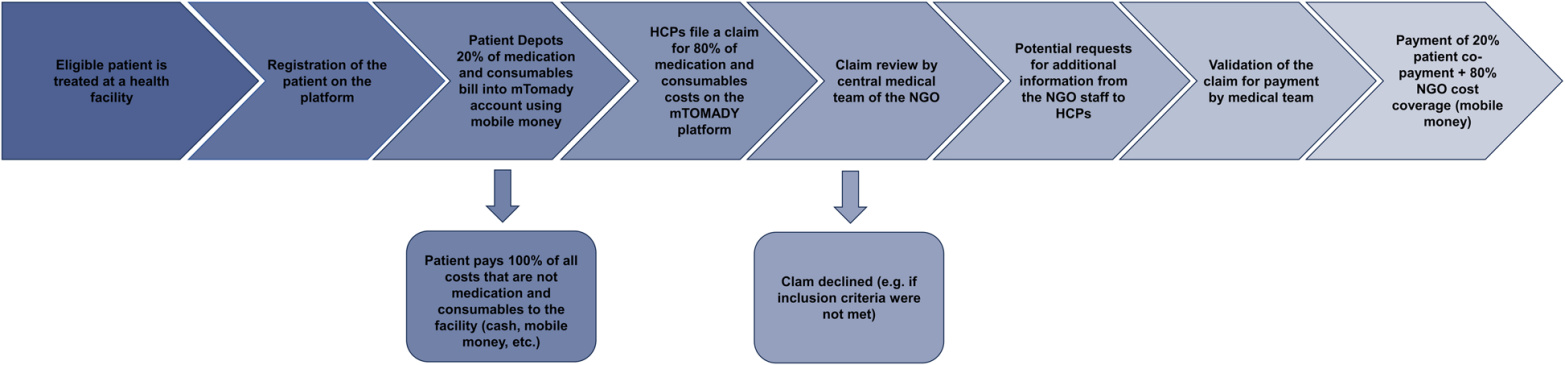
Process of patient and provider payments during a digital conditional cash transfer intervention for healthcare utilization

Upon transmission of the claim to the central mTOMADY server, 20% of total treatment costs were taken from the patient’s mTOMADY account. The claim was then forwarded to a central medical claims team at the implementing NGO, which checked the claim for consistency and missing data. If data in the claims were missing or inconsistent, the claims were returned to the center that had filed the claim, asking for clarification and additional details. If necessary, the medical claims team and the participating facilities communicated directly via phone or email—otherwise all communication was carried out via the mTOMADY platform. The 20% contribution of patients were put on the patients’ individual mTomady accounts and paid, together with the 80% contribution by the NGO directly to the health facilities via mobile money to the mobile money account of the facilities, bundled for all approved claims twice per month. Figure 2 below illustrates the flow of the claim filling and payment flow during the project.

## Data collection

### Inclusion and exclusion criteria

We conducted in-depth interviews with various stakeholders, including healthcare providers (HCPs), project implementation staff, policymakers, beneficiaries of the intervention, and non-beneficiaries. Non-beneficiaries were those individuals who were eligible for participation in the digital conditional cash transfer intervention (i.e., fell into one of the eligible patient groups and sought care at a participating health facility during the project time plan) but were not enrolled. Individuals were excluded if they were unable or refused to give informed consent, and if they were below 18 years of age (parents of patients falling in the children under 5 category were included). The detailed inclusion and exclusion criteria for each participant group are listed in Table 1 below.

**Table 1:** Study inclusion and exclusion criteria. Inclusion and exclusion criteria for participating in qualitative in-depth interviews related to unintended effects of a digital conditional cash transfer intervention for increasing healthcare utilization in Southern Madagascar, February 2021-July 2022

Participant group	Inclusion criteria	Exclusion criteria
Healthcare providers (HCPs)	- employed at partnering facilities during the intervention and in contact with the intervention	- not engaging with the intervention on a regular basis
Project implementation staff	- employed by the implementing NGO during the intervention - actively involved in the design, implementation, or administration of the intervention	- not involved in the design, implementation, or administration of the intervention for at least five months of the intervention’s duration
Policymakers	- employed by the Malagasy government in administrative responsibilities in the study region during the design, implementation, or running of the intervention	- not involved in the design, implementation, or running of the intervention
Beneficiaries	- received treatment cost subsidy through the intervention	- under 18 years of age - unwilling to or unable to participate in the interview (e.g. for health reasons)
Non-beneficiaries	- seeking care at a partnering health facility and during the intervention time frame and for a condition covered by the intervention and - did not receive treatment cost subsidy through the intervention	- under 18 years of age - unwilling to or unable to participate in the interview (e.g. for health reasons)

We developed separate interview guides for each participant group, enabling us to capture the unique experiences and perspectives of each participant group. The interview guides consisted of targeted questions covering several key areas: i) the participants’ experiences with the digital conditional cash transfer intervention, ii) specific aspects of the intervention related to its digital nature, iii) the overall implementation process of the intervention, iv) the (un)intended effects observed at both the facility and individual levels, and v) areas of concern or criticism. The English versions of all interview guides can be accessed in Supplementary File 1.

### Sampling

Distinct sampling methods were employed for each participant group in this study:

HCPs were recruited from the participating facilities either through phone calls or direct visits of research assistants at the facilities. We contacted at least one HCP per facility via telephone initially and then contacted further HCPs at the same facility on the day of the interview with the HCP initially contacted via telephone. We included a convenience sample of HCPs at each facility we approached, aiming to speak to at least 2 HCPs working in different functions at each facility (e.g. head doctors, head nurses, midwives, administrative clerks, etc.). We ensured that we spoke to at least 2 HCPs per facility from different functions through a snowballing approach limited to the facility: During the initial interview with the first HCP, we asked them to identify further colleagues who fulfilled different roles in the intervention for us to approach subsequently.

Facilities participating in this study were selected to represent a purposive sample of both high-performing and low-performing facilities, meaning facilities that enrolled a large (> 50%) and small (< 25%) proportion of eligible patients in the digital conditional cash transfer intervention during the intervention’s implementation. We included five high- and four low-performing facilities in our sample. We selected facilities that represented different levels of care (primary (three facilities) and secondary (six facilities), as well as a mix of public (three facilities), private (one facility), and faith-based (five facilities) care providers. The selection of the facilities was guided by geographical proximity of the facilities to correspond to the limited financial resources that were available for the study, the length of enrollment of facilities in the project (we aimed to capture those experiences in particular of facilities that had joint the project the earliest), and to reflect a mix of facilities as described above.

Project implementation staff members were recruited from the same high-performing and low-performing facilities as the healthcare providers, along with additional staff from the project’s management team in southern Madagascar and the administrative team in Antananarivo. Recruitment of project implementation staff was conducted via telephone or email contact. Project implementation staff were responsible for supporting the facilities during the intervention’s implementation, for example with the filling of claims. The additional individuals interviewed from the NGO’s administrative team were responsible for the claim validation process, intervention planning, and the financial administration of the intervention.

Policymakers were purposively sampled and contacted equally through telephone or email communication. Additionally, snowball sampling was used to identify other significant participants within this group.

Beneficiaries and non-beneficiaries were identified and approached in their respective communities with the assistance of community health workers. We used a purposive sampling approach and selected beneficiaries, and non-beneficiaries based on their proximity to partaking healthcare facilities in the rural Ampanihy district (Atsimo-Andrefana region) and the rural Tolagnaro district (Anosy region). At each health centre, we sampled one community located less than 10 km from the health centers and one community located more than 10 km away. We explained inclusion and exclusion criteria to community health workers to ensure they could help us sample community members according to these criteria. We employed a purposive sampling approach to ensure we sampled community members with pertinent experiences with the intervention.

### Data collection

A Malagasy researcher, fluent in native Malagasy dialects and French, and possessing expertise in qualitative data collection methods, was responsible for conducting the interviews. To enhance the credibility and depth of the data, the researcher underwent a comprehensive 3-day training covering various aspects of qualitative research methods, ethics, and the objectives of the study.

The purpose and objectives of the study were explained to all approached participants in their preferred language, Malagasy or French. The research assistant then obtained written informed consent from the participants. All qualitative interviews took place in settings chosen by the participants and were conducted in either local Malagasy dialogue or French, depending on the participant’s preference. With the participant’s consent, all interviews were audio-recorded and subsequently transcribed verbatim by the interviewing researcher.

Table 2 below illustrates the specific questions in the interview guides per participant group that were aimed at identifying unintended effects of the intervention. However, the guides were designed for semi-structured interviews. The interviewer adapted and probed into the question as individual conversations required it to explore the research objectives, of which unintended effects were one. Therefore, the whole transcriptions were used as a basis for this analysis and themes emerged beyond these specific questions.

**Table 2:** Questions used in qualitative interviews to identify positive and negative unintended effects of a digital conditional cash transfer intervention for enabling healthcare utilization in Southern Madagascar, February 2021-July 2022. The questions were established as guiding questions for semi-structured interviews and were therefore adapted during the conversations with participants to ensure rich individual data

Interview questions aimed at identifying unintended effects by participant group	
Non beneficiaries	• When you went to a healthcare centre, what about Tosik’aina was different from your expectations about Tosik’aina, if there is anything? • We saw many people who made use of Tosik’aina but also many who went to the hospital but did not use it. I can’t talk with all of them, that is why I am here with you and interested in your personal story. Can you tell me the story of how you needed healthcare but ended up not benefiting from Tosik’aina? • What were/are people discussing about Tosik’aina? • With which of the things said do you agree? With which do you not agree? • If you know individuals who used and/or individuals who were not able to use Tosik’aina, what did Tosik’aina change regarding who could go to the hospital, if anything? • What do you think about using mobile money to pay for health expenses instead of cash in the future? • Now you have provided me with many insights into your experience with Tosik’aina. If you were the designer of Tosik’aina and were to do it again, what are some key things that you would want to change? • And what would you want to keep?
**Beneficiaries**	• When you went to a healthcare centre, what about Tosik’aina was different from your expectations about Tosik’aina, if there is anything? • We saw many people who made use of Tosik’aina but also many who went to the hospital but did not use it. I can’t talk with all of them, that is why I am here with you and interested in your personal story. Can you tell me your story of how you came to use Tosik’aina? • How do you understand the payment that had to be made by you as the beneficiary when you went to the hospital and took part in Tosik’aina? • What were some things that did not work very well for you with Tosik’aina? • When you compare that period to the one that has passed since you learned about Tosik’Aina, what, if anything, has changed in your way of thinking about healthcare? • And what did Tosik’aina change in your life, if anything? • What were/are people discussing about Tosik’aina? • With which of the things said do you agree or disagree with? • If you know of people who have used and/or people who have not been able to use Tosik’Aina, what has Tosik’Aina changed regarding who could go to hospital, if any? • In other parts of the world, for example rural Bangladesh, using mobile money could be seen as a challenge for various reasons. One example could be misunderstanding how this technology works or an unreliable mobile network. Did you encounter similar difficulties in using Tosik’aina? • What do you think about using mobile money to pay for health expenses instead of cash in the future? • Now you have provided me with many insights into your experience with Tosik’aina. If you were the designer of Tosik’aina and were to do it again, what are some key things that you would want to change? • And what would you want to keep?
Healthcare providers (HCPs)	• I will speak with different healthcare providers who took over different roles within Tosik’aina. Today with you, I would like to hear your personal story with Tosik’aina. When you were introduced to Tosik’aina, what were your expectations? • Throughout the implementation of Tosik’aina, what about Tosik’aina was different from your expectations, if there is anything? • What effects did Tosik’aina have at your center? • What were effects of Tosik’aina that you did not expect? • Now, let us think through the journey of Tosik’aina, from the moment you got involved in the project until now: What problems did you encounter during this whole time with the implementation of Tosik’aina? • What do you think about the copayment that patients had to make? • How did Tosik’aina change the trust patients have in the health services you provide, if at all? • What did/do you hear people say about Tosik’aina? • Which patient groups did you identify that would have needed Tosik’aina most but were not able to access it for different reasons? • What could have been done to include these groups? • How did mTomady impact your workload and/or your workflow, if at all? • What do you think of the use of mTomady in your working context? • If you were the designer of Tosik’aina with all the knowledge you have now, what would be some key things that you would change about the intervention? • And what are key elements of the intervention that you would want to keep?
Policymakers	• Now, let us think through the journey of Tosik’aina, from the moment you first heard about the project until now: What problems arose in your area during this whole time with the implementation of Tosik’aina? • What effects of Tosik’Aina on collaborating/participating centers have you seen, if any? • What effects of Tosik’aina on centers without Tosik’aina did you see, if any? • How could Tosik’aina be adapted to align better with the health priorities on your agenda? • What were the effects of Tosik’Aina that you did not expect? • Which patient groups did you identify that would have needed Tosik’aina most but were not able to access it for different reasons? • When you were introduced to Tosik’aina the first time, what were your expectations? • Throughout the planning and implementation of Tosik’aina, what was different from your expectations, if there is anything? • If you were the designer of Tosik’aina with all the knowledge you have now, what would be some key things that you would change about the intervention? • And what are key elements of the intervention that you would want to keep?
Project staff	• I will speak with people who had different roles in Tosik’aina. Today with you, I would like to hear your personal story with Tosik’aina. When you started working on Tosik’aina, what were your expectations? • How did your perceptions of Tosik’aina change over the course of the implementation? • Now, let us think through the journey of Tosik’aina, from the moment you got involved in the project until now: What problems did you face during the implementation of Tosik’aina? • What could be or has been done to solve these challenges? • What helped to successfully implement Tosik’aina? • What were the effects of Tosik’aina that you did not expect? • Tosik’aina was intended to help especially very vulnerable groups. What do you think about how this goal was reflected in the design and implementation of Tosik’aina? • With the knowledge you have now after the end of Tosik’aina, which patient groups did you identify that would have needed Tosik’aina most but were not able to access it for different reasons, if there are any? • What do you think about the use of mTomady for Tosik’aina? • How appropriate is technology in the context you work in? • If you were the designer of Tosik’aina with all the knowledge you have now and were to do it again, what would be some key things that you would change about the intervention? • And what are key elements of the intervention that you would want to keep?

### Data analysis

Trained interpreters translated the interview transcriptions into English for analysis. Before transcription and translation, all identifying information was anonymized by the researcher who conducted the interviews.

To ensure accuracy and validity, two native Malagasy speakers conducted random spot checks, comparing the transcripts and translations to the original recordings. All data were securely stored in a digital format within a password-protected database.

Using an inductive approach based on reflexive thematic analysis [20], 4 researchers (A.N., M.F., O.R., and D.S.) individually coded the interviews. All researchers separately coded each transcript and then compared their coding results to ensure consistency. After discussing the initial coding, M.F. did an in-depth coding for this research objective. The refined codebook and the ongoing interpretation of results were repeatedly discussed within the research team to check consistency between the researchers’ perspectives. Codes and themes were created as they emerged from the data. The codebook was continuously expanded, and re-organised as additional codes emerged, or codes were adapted. Themes were equally created as they emerged from the data and where appropriate, themes were further organized into overarching themes. Regular meetings were held among the researchers to discuss emerging themes and reflect upon them. If necessary, coding was revised accordingly. All analyses were conducted in NVivo version 12 [21].

**Fig. 3:**
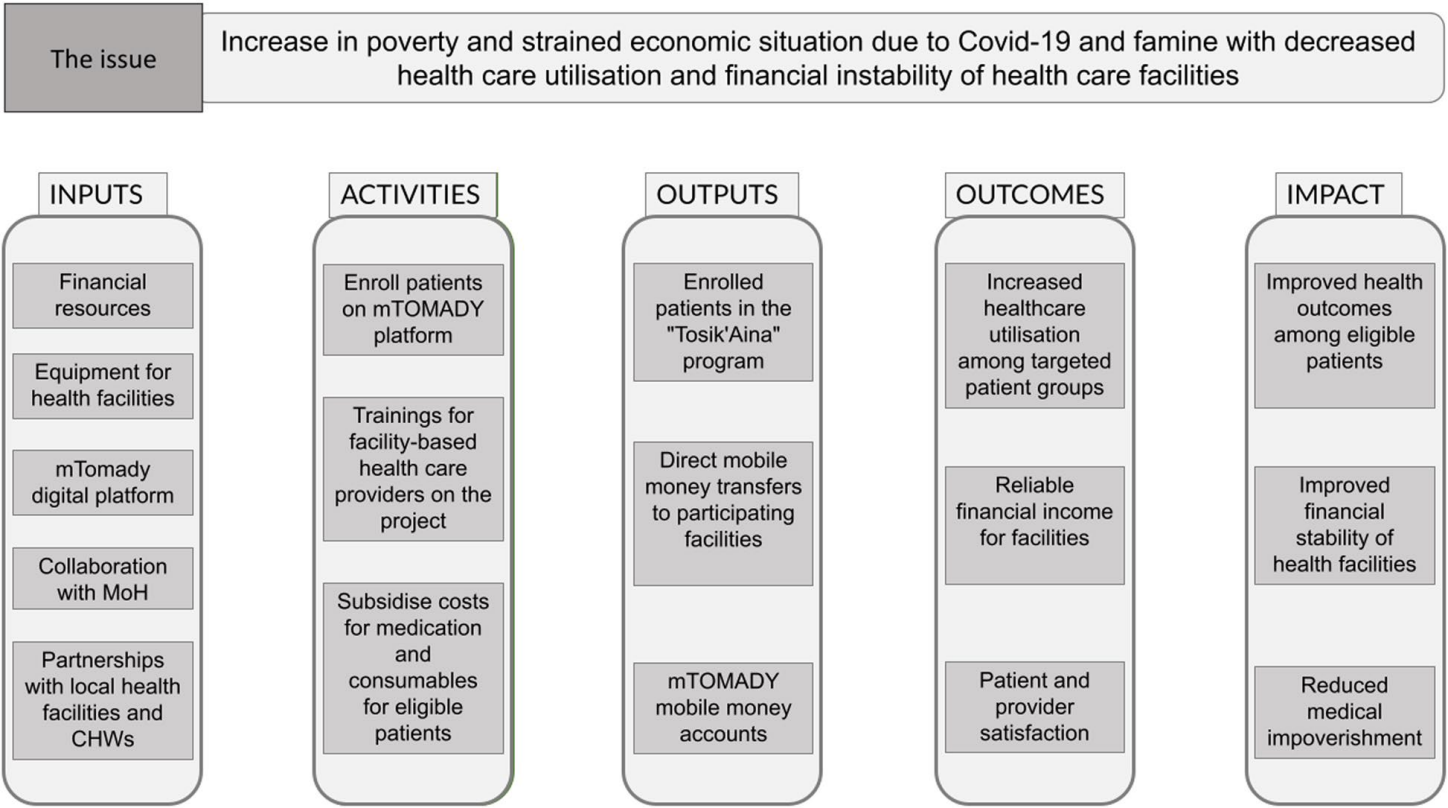
Theory of change for a digital conditional cash transfer intervention for healthcare utilization. MoH = Ministry of Health, CHWs = Community health workers. The Theory of change is based on 3 underlying assumptions: 1) Providing a subsidy for medications and consumables will increase healthcare utilization among targeted patient groups. 2) Covering 80% of the expenses will improve the revenue and financial stability of healthcare institutions. 3) The mTOMADY digital platform will facilitate enrollment, claim submission, verification, and fund transfers effectively

For the purpose of this study, we defined intended effects as any consequences of the intervention that were envisioned as outputs, outcomes, or impacts of the project included in the intervention’s initial theory of change which is represented in Fig. 3 below. The intended effects of the intervention were i) an increase in healthcare utilisation among targeted patient groups, leading to improved health outcomes among eligible patients, ii) reliable financial income for facilities, leading to an improvement in their financial stability, iii) increased patient and provider satisfaction, and iv) a reduction of medical impoverishment in the target population.

Unintended effects were conversely defined as any consequences other than these intended consequences envisioned under the original theory of change.

### Ethics approval and consent to participate

This research was carried out in compliance with the Declaration of Helsinki. The study received ethical approval from the University of Heidelberg Ethics Committee (Heidelberg, Germany), registered under the number S-982/2021. In addition, we obtained formal approval to conduct this study from each district health office (a. regional sub-division of the Malagasy Ministry of Health) in which interviews were conducted. Written informed consent was obtained from each interview participant prior to the interviews.

## Results

In total we drew on data from 63 interviews lasting between 30 min and 1.5 h with an average interview time of 47 min. The data collected in response to the questions listed in Table 2 above on average responded to 28 min of the interviews (range: 18 min to 1 h). Ten interviews were conducted with project implementation staff (3 women, 7 men; average age: 31.5 years (range: 27–44 years), 22 with healthcare providers (11 women, 11 men; average age 37.5 years (range: 24–58 years), 17 with beneficiaries (15 women, 2 men; average age: 40.9 years (range: 19–56 years), nine with non-beneficiaries (5 women, 4 men; average age: 43.1 years (range: 18–73), and six with policymakers (3 women, 3 men; average age 49.2 years (range: 46–55); age was missing for one policymaker).

Of the 22 healthcare providers four were nurses, one was a midwife, three were the heads of health centres, 11 worked as pharmacists or administrative clerks, and the remaining four had other medical professions, e.g. physicians’ assistants. 12 healthcare providers were interviewed in Anosy region, three in Androy region, and seven in Atsimo-Andrefana region. Nine healthcare providers were working at primary and thirteen at secondary facilities. Five healthcare providers were employed by private health centres, ten by faith-based health centres, and seven by public health centres.

For policymakers, two were based in Anosy region, four in Atsimo-Andrefana region, and one in the capital city Antananarivo in Analamanga region. Out of ten project staff members who were interviewed, three were based in Antananarivo, three worked in Anosy region, and four in Atsimo-Andrefana.

Among beneficiaries, most had been eligible to be included in Tosik’aina under the “potentially life-threatening illness” category (12 out of 17), four were seeking maternity care, and one family was seeking care for a child under five years of age. This mirrors the quantitative care-seeking patterns of the intervention, where most patients were seeking care for “potentially life-threatening illnesses” and maternity care. Nine of the respondents who had benefited from the intervention stated their profession as “farmers”, eight said they had no occupation. All non-beneficiaries would have fallen into the category of “potentially life-threatening illness”. Of nine non-beneficiaries, four said they had no occupation, one participant stated that he was retired, and the remaining four gave their professions as “farmers”.

Our analysis revealed six key unintended consequences, three positive and three negative ones, illustrated in Table 3 below.

**Table 3:** Positive and negative unintended effects. Positive and negative unintended effects of a digital conditional cash transfer intervention for enabling healthcare utilization in Southern Madagascar, February 2021-July 2022. In brackets: participant groups who mentioned the respective effects. B = Beneficiaries, HCP = HealthCare Providers, NB = Non-Beneficiaries, PM = Policymakers, PS = Project Staff)

Unintended effects of the digital conditional cash transfer intervention	
*Positive*	*Negative*
**Quality of care (HCPs, PS, PM)** *Increased guideline adherence* *Investments in infrastructure and medical equipment*	**Increase in patients exceeding facilities’ capacity (HCP, B, PM)**
**Interpersonal relationships (B, HCPS, PS)** *Improvement in patient-provider relationships* *Improvement of the collaboration within the facility*	**Increase in costs of care (NB)**
**Digital nature of the intervention (B, HCP, PS, PM)** *Digital skills development of facility staff* *Increase in trust in mobile money*	**Cases of hospital imprisonment (B, NB, PS)**

### Positive unintended effects

Positive unintended effects of the intervention can be grouped into 3 overarching categories: unintended effects related to the quality of medical care provided, unintended effects related to interpersonal relationships, and unintended effects related to the digital nature of the intervention.

### Quality of care

#### Increased guideline adherence

A noteworthy positive effect of the intervention that was reported by several participants was a perceived increase in the quality of care provided to patients. For example, one participant reported an improved adherence to the malaria treatment guideline for children in the hospital, after they had received several requests for clarification from the medical team of the NGO. Additionally, the administrative processes of the digital conditional cash transfer intervention had a positive impact on the documentation of the treatments provided to patients.

*“[The intervention] has improved the quality of our healthcare to get out of the bad routine. Before we did not master the observation form, that is the medical form of the patient. But when there was the [claims system], it improved the quality of filling in the form used for a patient” (HCP 16, nurse, male, 39 years old).*

*“Before, here in the south, when they made a diagnosis, it was done approximately, […] And I saw that the capacity of the doctors increased, and they know the patients better. This is a positive impact on the process applied. (Project staff 6, male, 30 years old).*

#### Investments in infrastructure and medical equipment

Several HCPs expressed that the intervention increased the financial stability of the facilities, as they could rely on patient costs being paid in full through the intervention, which allowed them to pay salaries on time and generate a continuous supply of medication and consumables for which suppliers relied on a pre-payment system. While this was a consequence envisioned under the original theory of change of the project, the generation and use of surplus income was not. Several participants reported that this income allowed the facilities to invest in medical infrastructure and equipment, leading to long-term positive effects on the quality of medical care.

*We bought a lot of things, even the oxygen concentrator that we brought in. So, we were able to renew our equipment with the money we earned at that time because our sales increased enormously. (HCP 5, physician assistant/other medical staff, female, 28 years old).*

### Interpersonal relationships

#### Improvement in patient-provider relationships

Several HCPs reported an improvement in the patient-provider relationships during the intervention. They expressed that the feeling of support patients received through the presence of financial aid, but also the aid of HCPs that often guided patients through the intervention’s enrolment process improved the relationship and created a sense of trust between patients and HCPs.

“The unexpected results that I saw was that it created a relationship of trust between the patient and the facility.” (Project staff 4, female, 29 years old)

*Yes, and I told them: “Thank you very much for the help you gave me, for saving my life. I was breathless, I could not breathe, as soon as you gave me medicine, I was healthy.” (Beneficiary 9, female, 66 years old).*

*“In this regard, perhaps the change is that the patient realized that he is not alone in his difficulties, that there is someone who supports him, who helps him” (HCP 3, nurse, female 44 years old).*

In part, this positive effect may further be attributed to an increase in transparency of healthcare payments and financial accounting within the facilities. Patients knew exactly how much fees were being charged, and facilities were forced to do detailed bookkeeping and financial accounting, increasing trust in the system.

*“mTOMADY has promoted transparency within the facility. The prices are very accurate and everything that is written down is well followed up according to the payment made by the patient, which decreases corruption. It is also very clear to the patient what they have to pay and what has been taken care of. It is good in terms of a transparency system.” (Project staff 6, male, 30 years old).*

*“I like the way of working: taking photos, using the ID card, *etc*., to justify that there is no scam, but what we do is true, it is clear, and I like it”. (Beneficiary 8, female, 36 years old).*

#### Improvement of the collaboration within the facility

Several participants further expressed a positive impact of the digital conditional cash transfer intervention on collaboration within the health facility. The administrative processes associated with the intervention required increased cooperation and communication between different staff members at participating facilities, which fostered a sense of communal work and community responsibility.

*“It was great because we were able to communicate with each other, to help each other even if we did not know each other, even if they were strangers. We did not know each other, but it was the work that brought us together. It was great. About the staff, the collaboration here was wonderful.” (HCP 4, pharmacist, female, 25 years old).*

It must be noted however that this was not a universal effect and that some, albeit fewer, participants reported a diverging view:

*“For the case of [this facility] only, there was a big problem because even if the doctor or the midwife and so on have already selected the people who come, the pharmacist rejects them because he is suspicious that the money will not be paid. Because the pharmacist in the hospital is an external provider” (Project staff 10, male, 44 years old).*

### Digital nature of the intervention

#### Digital skills development of facility staff

HCPs noted that the digital administration of the intervention had significantly increased their digital literacy and prowess, making them feel more equipped for their current work and more confident about potential future employment opportunities.

*“There is also the use of the tablet, the application that is inside, the system used. It was all new to me but thanks to the existence of the intervention I was able to learn it. These are advantages, I was able to acquire knowledge and courage. […] About technology, if for example, my knowledge was 30% before, when there was mTOMADY it went up to 60%. Because there were new things that I did not know before and then I mastered. “(HCP10, physician assistant/other medical staff, male, 24 years old).*

#### Increased trust in mobile money

Similarly, beneficiaries of the intervention, as well as HCPs reported an increase in trust in digital technologies, especially in mobile money. The main advantage that they perceived was that mobile money was something that could not be stolen or taken from them.

*“If you put the money in your pocket, someone could steal it, and the money is lost, and it is a loss. But if you put it in the sim card, even if the sim card is lost, you can recover.” (Beneficiary 8, female, 36 years old).*

*“It suits me because it is my SIM card, for example the SIM card is damaged, you can always get it. If you keep the money in your hand, it can be lost, and it will be a loss.” (Non-beneficiary 9, female, 36 years old).*

### Negative unintended effects

#### Increase in patients exceeding facilities’ capacity

The most commonly reported negative unintended effect for facilities was that the increase in patient numbers exceeded their capacities to deal with these patients, including the workload for HCPs and the physical capacities of the facility.

*“The problem I faced: I was really tired, really tired. There is nothing to say but that, I was tired, my body was tired, my mind was tired, I was missing food. […]. The whole team involved in [the intervention] lost kilos! (HCP 4, pharmacist, female, 25 years old).*

*“But it is the employees who are the victims because their workload increased.” (HCP 15)”*

*“Our patients increased, not by 100% but maybe 200%. Our life was not going to support the work because we were very tired but what to do, we had to face.” (HCP 8, physician assistant/other medical staff, female, 35 years old).*

*“When there is Tosik’Aina we are happy and many of us go to the hospital because we are happy that Tosik’Aina is coming. (Beneficiary 6, female, 36 years old)”*

For several facilities, this resulted in the need to reinvest some of the money made to maintain or refurbish their facilities.

“There were some materials that were lost due to the high number of patients, beds, mattresses” (HCP 13, physician assistant/other medical staff, female, 24 years old)

*“In terms of equipment, we were really annoyed because there were many patients and the equipment was insufficient. There was this insufficiency but there were also many rural people who did not respect things, and several materials were damaged. The mattresses, the beds were damaged, and they left them there, everything was almost damaged.” (HCP 10, physician assistant/other medical staff, male, 24 years old).*

Sometimes this increase in patient numbers also meant that patients were discharged early, which might have had negative consequences for their health status in the long run. This was perceived as worrying by several participants.

“It was so full that some were sent home, even though they were not completely cured but safe.” (HCP 10, physician assistant/other medical staff, male, 24 years old)

*“Yes, it exceeded too much. If you go through the courtyard now, you will see people sleeping […] on the floor, without mats or mattresses or anything else.” (HCP 15, head of medical facility, male, 45 years old).*

### Increase in costs of care

An important unintended consequence of the intervention was an increase in costs of care at facilities, as reported by some participants. According to them, some facilities increased their prices after the initiation of the intervention, knowing that the increase would be covered through the NGO’s financial support system. However, the amount of money paid by patients was still a sum relative to the total amount, which meant that costs not only increased for the NGO but also patients, potentially reducing the positive effect of decreasing the cost of care on healthcare utilization and medical impoverishment.

*“Since the existence of [the intervention], it has increased. In the past, an appendicitis surgery was 600,000 Ariary [132 United States Dollars]. Now, because of the medicines supported by [the NGO], it has risen to 1,600,000 Ariary [352 United States Dollars]. This is the truth I am telling you. This is the truth. It has become a problem for us as a client. […] So, it is because of the existence of the help provided by [the NGO] that the price has increased there.” (Non-beneficiary 7, male, 45 years old).*

### Cases of hospital imprisonment

Some facilities struggled with financial loss when claims were declined by the NGO’s claims team (e.g., because of errors in the filing of the claim or because the patient was ineligible), particularly if the patient had already been discharged and the facility had no way of recovering the costs from the patients. Some participants indicated this might have resulted in the patients being kept in the hospital for a longer time than necessary and potentially against their will, increasing the costs incurred by patients (e.g., for the room) and infringing on their personal rights.

“*When there was a sick person, he was kept there like a prisoner*” (Beneficiary 4, male, 31 years old).

## Discussion

Our study revealed two key findings related to unintended consequences of the digital conditional cash transfer intervention.

Firstly, most of the unintended consequences were positive consequences related to the dimensions of quality of care, interpersonal relationships, and the digital nature of the intervention. Secondly, the intervention also caused three important negative unintended consequences: i) Increase in patients exceeding facilities’ capacity, ii) Increase in costs of care, and iii) Cases of hospital imprisonments.

Through the design of the digital conditional cash transfer intervention, necessitating the interaction between different levels of hospital staff and the review of each case by an independent claims team, the project had positive influences on the quality of care that patients received and likely ultimately improved their health outcomes. This ties in with findings from other countries in SSA, demonstrating an improvement in healthcare services, including laboratory services and family planning services with external supervision and feedback [22–24]. Further, evidence suggests that supervision might also increase HCP job satisfaction and motivation [22–24]. Additionally, the intervention allowed hospitals to invest in infrastructure and medical equipment, likely increasing the quality of patient care further. However, long-term challenges may arise from these investments in the absence of centralized leadership and strategic oversight. Specifically, a lack of coordinated planning across facilities can lead to inefficient allocation of resources, with certain facilities overinvesting in some types of equipment and underinvesting in others, potentially increasing inefficiencies within the health system.

Participants remarked on an improvement in interpersonal relationships, both between HCPs working at the same facility but also between patients and providers. This improvement was notably down to two factors: patients felt more supported and aided by HCPs in accessing care and the increase in transparency of patient payments and financial transactions for healthcare. This latter aspect should be of particular note to future cash transfer interventions, particularly those using digital technologies, which should proactively promote transparency in their design and implementation, especially as good patient-provider relationships have been shown to improve patient treatment adherence and quality of life [25–29]. These findings are also relevant for broader policy change in low-and-middle income health systems. Promoting transparency in the financial administration of health centres and health financing and thus increasing patient trust is a key step policy makers can take to build the long-term resilience of health systems in low-and-middle income countries.

The perceived increase in digital skills among healthcare providers is an important consequence to note, as a lack of digital literacy is often a key barrier to the successful implementation of digital health interventions in SSA highlighted by evidence from Ethiopia and South Africa [30–33].

Beneficiaries of the intervention in our sample reported an increase in their trust in mobile money use. Given the effectiveness of mobile money, not only in promoting the use of healthcare services and health outcomes, including the adherence to tuberculosis treatment and the update of vital maternal health services [34, 35], but also potentially its role in national development and financial inclusion on a larger scale [36], this is an important effect to note and foster in similar interventions. Broader health system financing interventions and policies should equally consider the ongoing expansion and reach of mobile money and its accessibility for social strata of lower socioeconomic status and leverage the opportunities the technology poses for the expansion of access to healthcare services as evidenced by successful interventions in Rwanda and Kenya [37].

However, our study also revealed several negative unintended consequences associated with the intervention.

Firstly, the increase in patients exceeding facilities’ capacity is a factor that should closely be considered by any intervention aiming at increasing healthcare utilization, as the limitations of facilities, both in physical capacity and in staff time and workload, can have important negative repercussions on patient outcomes. In our study, providers reported discharging patients early to make room for others. Discharging patients early might negatively impact patient outcomes, even though evidence of this effect from low- and middle-income countries is limited [38]. In light of this, future implementers should anticipate such effects on health facilities and proactively prepare facilities for the increase in patient numbers, e.g. through investment in additional equipment or auxiliary staff.

Further, exceeding facilities’ capacities to manage patients, the necessity to learn new skills for the digital administration of the project, as well as the external review of each claim by a claims team were perceived to increase provider workload. Given that the workload for HCPs across SSA often already exceeds providers’ capacity and is a key risk factor for HCP burnout in the region [39, 40], this is an essential negative unintended effect to consider. As evidence further suggests that high provider workload might negatively impact patient-provider relationships and the quality of care provided, the negative consequences of overcharging facilities’ capacities might negate important positive consequences of the intervention [40, 41].

Secondly, participants reported an increase in overall costs of care due to the presence of the NGO intervention. While this was only reported by one participant, we felt it was an essential unintended consequence to highlight, as it clearly contradicts the intervention’s goals of reducing financial barriers to care. Our interpretation of the observed price increases is that they were likely related to diagnostic services or interventions, which were not covered by the predefined price lists and thus not subject to the cost monitoring processes implemented as part of the intervention. We can also not exclude that facilities charged patients additionally for services but did not report these costs on the claims filled with the implementing NGO to circumvent the price control mechanisms put in place by the NGO. As patients paid a percentage price of the overall costs as their contribution, this overall price increase also increased their share and thus direct medical costs at the point of care. As an increase in costs at the point of care negatively impacts patients’ healthcare utilization and high out-of-pocket costs for patients at the point of care are a key driver of catastrophic health expenditure [42–44], designers and implementers of future (digital) cash transfer interventions for healthcare need to foresee and prevent such an increase in costs driven by the presence of an intervention, particularly when the goal of the intervention is to reduce financial barriers to care. Possible strategies to do this could include negotiating maximum prices with healthcare providers or adding additional financial risk protection mechanisms for patients who cannot afford the 20% contribution.

Lastly, our study suggested that the intervention’s design could have led to cases of hospital imprisonment. Cases of hospital imprisonment are common in Sub-Saharan Africa [45, 46] and there is no reason to believe that this intervention increased their occurrence but rather highlighted an already prevalent practice.

These cases occurred when patients had to wait for their claims to be fully processed by the claims team because hospitals feared a claim might be declined and the hospital would subsequently lose income. Any delays in the claims processing thus led to patients staying in the hospital longer and consequently an increase in patient costs (e.g., for per diem rates for hospital accommodation), with negative consequences on patients’ financial situation, especially as such costs driven by longer hospital stays were not covered through the intervention, which only covered medication and consumables. As such, the positive aspects of the external validation of all claims might have been seriously impacted by the negative consequences on patient costs related to the delay caused by the claim validation process, undermining the overall goal of the intervention of easing the financial burden associated with medical care.

It is important to acknowledge that, given unreliable hospital funding from the government level, especially in rural areas in Madagascar, hospitals are particularly reliant on out-of-pocket payments from patients and highly vulnerable to missed or lost payments [3, 19]. Designers and implementers of similar interventions therefore need to envision strategies to monitor and prevent cases of hospital imprisonment, while taking into account health facilities’ financial vulnerability. Such strategies could include the change of funding mechanism, providing facilities with an unconditional sum of money to offset income losses when cases are declined. Alternatively, incorporating additional digital technologies, such as artificial intelligence, could be explored to accelerate the claim validation process.

Overarchingly our findings underline the need for future research and investigation into unintended consequences of cash transfer interventions, be they digital or analogue, in order to identify common as well as diverging experiences. Additional research into potential mitigation strategies for the negative consequences described in our study would be valuable.

Despite its important findings, our study has several important limitations. Firstly, the study drew on data collected from selected facilities that benefited from the intervention. The facilities were purposely sampled to include facilities that were in the project for a long time, as well as for a shorter period, and to include facilities that performed well (i.e., included a lot of patients in the project), as well as those that did not. However, given that we only included certain facilities, we might have failed to capture all unintended consequences of the intervention or important nuances of them. Similarly, interviews with beneficiaries of the intervention were limited to a convenience sample of a few communities in rural Southern Madagascar and not representative of all individuals that benefited from the digital conditional cash transfer intervention.

Secondly, the data were originally collected in Malagasy and translated into English for analysis. This might have introduced misunderstandings or mistakes in how the data were interpreted in the process. We did, however, take a variety of steps to mitigate this. All data were transcribed by a trained, professional interpreter and two native Malagasy speakers who were part of the research team conducted random spot-checks of the translations against the original audio recordings of the interviews to ensure their validity. Additionally, research staff met regularly to discuss and alleviate any unclarities in the data or possible translation errors.

Third, as this is a purely qualitative study, we have no ability to evaluate the respective magnitude of different unintended effects. This is particularly relevant as we found positive and negative unintended effects that are contradictory, such as the increase in patient numbers at the facilities, which has both positive effects on facility income, but also negative effects on provider workload and the early discharge of patients. Given the design of our study, we cannot ascertain which effects dominated and might thus require relatively more attention from future implementers and policymakers.

## Conclusions

Overall, our study carries important lessons for designers and implementers of future (digital) cash transfer interventions with a similar scope and context: the process of external supervision of each claim increased both the quality of healthcare and the transparency of financial transactions for healthcare, in turn improving patient-provider relationships. Implementers and policymakers should invest in transparency in healthcare financing and in leveraging the technology of mobile money for healthcare payments to build on this positive effect.

The negative unintended consequences revealed in our study, such as hospital overcrowding, increased costs of care, and hospital imprisonments, underscore the need for careful consideration and proactive mitigation strategies when designing and implementing cash transfer interventions aimed at increasing healthcare utilization. Such strategies could include the investment in facility infrastructure and staff, the negotiation of price caps, or the structuring of payment systems in a way that prevents the occurrence of hospitals imprisonment.

## Supplementary Information

Supplementary Material 1.

## Data Availability

The datasets generated and analysed during the current study are not publicly available due the sensitive nature of qualitative data collected but are available from the corresponding author on reasonable request.

## Abbreviations

CHW: Community Health worker
CSB: Centre de santé de base
DFM: Doctors for Madagascar
GDP: Gross domestic product
HCP: Healthcare provider
NGO: Non-governmental organisation
OOP: Out of pocket
PPP: Purchasing power parity
SIM: Subscriber Identity Module
SSA: Sub-Saharan Africa
USD: United States Dollars
