# Supplementary material for: A qualitative analysis of unintended effects of a digital conditional cash transfer intervention to encourage healthcare utilization in Southern Madagascar
Source: BMC Health Serv Res. 2025 Feb 5;25:206. doi: 10.1186/s12913-025-12354-z (PMC11796226; doi:10.1186/s12913-025-12354-z)
Supplement: Supplementary file 1 — Supplementary Material 1. [file 12913_2025_12354_MOESM1_ESM.pdf]

Participant identifier: ________________________ Date: ___________________

Interview Guide IDIs Non-beneficiaries

I1	Interview Number	_______
I2	Name of Interviewer	___________________________________
I3	Interview Date	___ . ___ . ______ (dd.mm.yyyy)
I4	Time Start	___:___
I5	Time End	___:___
I6	Outcome of Interview	☐ Complete<br>☐ Incomplete, reason:<br>_______________________________
I7	Was the interview interrupted? (another person coming in, noises etc.)	☐ No<br>☐ Yes - by whom, how and why?<br>_______________________________

Respondent profile

R1	Gender	☐ Male<br>☐ Female<br>☐ Other
R2	Area	☐ Anosy<br>☐ Atsimo-Andrefana<br>☐ Other: ___________________

Participant identifier: ________________________ Date: ___________________

R3	Fokotany	____
R4	Age	____ years old
R5	Occupation	____________________________
R6	Potential beneficiary group (= group the participant would have belonged to, had she/he used Tosik'aina; referring to time of intervention)	☐ Guardian of child <5 years<br>☐ Pregnant woman<br>☐ Casualties<br>☐ Potentially mortal disease

Participant identifier: ________________________ Date: ___________________

Thank you very much for agreeing to participate in an interview for our study on Tosik'aina. Our aim in this part of the study is to understand how the program was perceived by the **non-beneficiaries**, what worked well, what didn't. Our goal is to improve similar interventions in the future to make them more accessible and beneficial.

I would now like to address a few general things about the project and the interview itself before we really get into it: First, we are interested in your personal experiences, opinions, and thoughts. There are no right and wrong answers, it is all about your personal experiences, views, and perceptions. Feel free to share whatever comes into your mind.

As we described in the information sheet, participation is anonymous. Your statements will be kept completely confidential and thus whatever you say will not have any influence or consequence on you and your employment. The data will be stored and analysed anonymously. It will not be possible to trace any statements back to you as an individual. This is to reassure you that you may also share critique. Please be also reminded that your participation is entirely voluntary. You can refuse to answer specific questions that you do not want to answer and you can stop the interview at any time. Please also let us know if you do not want to answer a specific question.

During the interview, I will be taking notes. This will help me and the research team to better understand the context of the statements, so please do not be confused about this. Also, I would like to ask for your permission to audio-record this interview so that we can stick to your original statements and not misinterpret things that you say. Is this ok for you?

[WAIT FOR PERMISSION AND NOTE IN FIELD NOTES]

What I just explained is also written in the consent form which I would like to ask you to sign.

The interview will last for about 45 minutes.

Do you have any further questions regarding the background of this study or the upcoming interview?

[LEAVE TIME FOR UPCOMING QUESTIONS]
Great! Then let us start

[*Reminder: the leading research questions for this interview are:*

1) ***What made it easy or hard for people to profit from Tosik'aina?***
2) ***What role did it play that Tosik'aina was based on the use of mobile phones and mobile money?***
3) ***What effects that were not planned did Tosik'aina have?***]

[START RECORDING]

Thank you for participating in this interview! Today is the [DATE] and this is the interview with [patient identifier]. I would now like to talk with you about your experiences with visiting a hospital related to the Tosik'aina programme, which took place during the last two years.

Participant identifier: ________________________ Date: ___________________

Topic 1	*Ice breaker questions*
1.1	As said, this interview is about your personal experiences and opinions related to healthcare and financial support for it. This is why I would love to first get to know how you are doing at the moment?
1.2	Let us think back to the last two years, now: How did the pandemic and the famine affect whether you went to see a doctor or other healthcare personnel or not when you needed one, if at all?<br><br>Probes:<br>- Which influences had changes in your community life?<br>- What role did the fear of infection play, if at all?<br>- What role did the government lockdowns play, if at all?<br>- What role did changes in available public transportation options play, if at all?<br>- What role did the costs play, if at all?<br>- How did your use of traditional care vs. healthcare in the CSB or hospital change, if at all?<br>- How did changes in the availability of money influence your use of healthcare, if at all?

I would now like to turn to the Tosik'aina program.

Topic 2	*Tosik'aina usage*
2.1	Have you heard about Tosik'aina?<br>*[If the interviewee does not know about Tosik'aina, provide the following definition:]* Tosik'aina took place in the last two years in many communities in the South of Madagascar, including yours. When pregnant women, children under 5, accident victims, or people with life-threatening diseases had to go to the hospital, the NGO Doctors for Madagascar payed 80% of the costs for drugs and consumables to decrease the costs for the treatment for the individual.<br><br>*If heard before:* What were your expectations when you heard about it?<br><br>OR<br><br>*If not heard before*: What are your expectations about the programme?
2.2	When you went to a health care centre, what about Tosik'aina was different from your expectations about Tosik'aina, if there is anything?
2.3	We saw many people who made use of Tosik'aina but also many who went to the hospital but did not use it. I can't talk with all of them, that is why I am here with you and interested in your personal story. Can you tell me the story of how you needed healthcare but ended up not benefiting from Tosik'aina?

	Probes:<br>- *Decision-making - process:* *[only if participant had heard about Tosik'aina before this interview]* You just told me the story of how you came to not use Tosik'aina. I would like to understand more about your thoughts at that time. What was in your mind when you would have been able to use Tosik'aina?<br>- *Decision-making - influences:* With whom did you talk about it when you came in contact with Tosik'aina?<br>- *Accessibility:* [*adapt this probe to the statements made by the participant*] Why was it difficult for you to use Tosik'aina?
2.4	How do you understand the payment that had to be made by the beneficiary in Tosik'aina?<br><br>*[If the interviewee does not know about the co-payment, provide the following definition:]* When a patient went to the facility and was registered as beneficiary of Tosik'aina, he or she had to pay something after he or she had received health care service.<br><br>*[If the participant still cannot speak about his perceptions of the co-payment, you may provide this definition:]* Through Tosik'aina, an NGO paid for 80% of the costs at the centre, but the patients still had to pay 20% of those costs. This part that the patient has to pay is what we are referring to here.<br><br>Probes:<br>- What do you think about this co-payment?<br>- Where did you hear about the details of the co-payment?<br>- Who are the winners of the co-payment?<br>- Who are the losers of the co-payment?<br>- Was this co-payment part of the reason why you did not use Tosk'aina?
2.5	What were/are people discussing about Tosik'aina?<br><br>Probes:<br>- What did the chef Fokotany tell you about Tosik'aina?<br>- What was discussed in Fivorianas?<br>- *[If a woman]:* What did you talk about concerning Tosik'aina in the women groups?<br>- Which words come into your mind when you think about Tosik'aina in your community?<br>- *Specific rumours:* What were positive and negative things about Tosik'aina that they mentioned?
2.6	With which of the things said do you agree? With which do you not agree?

2.7	If you know individuals who used and/or individuals who were not able to use Tosik'aina, what did Tosik'aina change regarding who could go to the hospital, if anything?<br><br>Probes:<br>- *Socioeconomic status:* What role did it play how much money people have in whether they were able to use Tosik'aina?

Because Tosik'aina was based on your mobile phone, I would like to ask you some questions about the technology aspect of it.

Topic 3	mTomady usage
3.1	What does mobile money mean to you?<br><br>Probes:<br>- When do you use mobile money?<br>- How often do you use mobile money?
3.2	What do you think about using mobile money to pay for health expenses instead of cash in the future ?<br><br>Probes:<br>- What would push you to use mobile money?<br>- What would prevent you from using mobile money?

Ending the interview

3.3	Now you have provided me with many insights into your experience with Tosik'aina. If you were the designer of Tosik'aina and were to do it again, what are some key things that you would want to change?
3.4	And what would you want to keep
3.5	Is there anything I should have asked you but didn't?<br><br>Is there anything else you would like to tell us?
3.6	________<br>*[ask question suggested by participant if applicable]*

Participant identifier: ________________________ Date: ___________________

**Interviewer comments:**
What are your impressions of the interview? Did you observe something special? Was there anything surprising / new for you? Was anything different than in other interviews?
Were there any problems with the interview guide or the related documents?<br>Was the place for the meeting point chosen well? Is there anything else we could consider changing?

**Reflection**
How do you think this interview went? How difficult was this interview for you?<br>How much do you feel that you affected this interview? What else would be important to add?

Participant identifier: ____________________ Date: ________________

Participant identifier: ________________________ Date: ___________________

Interview Guide IDIs Beneficiaries

I1	Interview Number	_______
I2	Name of Interviewer	______________________________________
I3	Interview Date	___ . ___ . ______ (dd.mm.yyyy)
I4	Time Start	___:___
I5	Time End	___:___
I6	Outcome of Interview	☐ Complete<br>☐ Incomplete, reason:<br>__________________________________
I7	Was the interview interrupted? (another person coming in, noises etc.)	☐ No<br>☐ Yes - by whom, how and why?<br>__________________________________

Respondent profile

R1	Gender	☐ Male<br>☐ Female<br>☐ Other
R2	Area	☐ Anosy<br>☐ Atsimo-Andrefana<br>☐ Other: ____________________

Participant identifier: ________________________ Date: ___________________

R3	Fokotany	____
R4	Age	____ years old
R5	Occupation	____________________________
R6	Beneficiary group (referring to time of intervention)	☐ Guardian of child <5 years<br>☐ Pregnant woman<br>☐ Casualties<br>☐ Potentially mortal disease

Participant identifier: ________________________ Date: ___________________

Thank you very much for agreeing to participate in an interview for our study on Tosik'aina. Our aim in this part of the study is to understand how the program was perceived by the beneficiaries, what worked well, what didn't. Our goal is to improve similar interventions in the future to make them more accessible and beneficial.

I would now like to address a few general things about the project and the interview itself before we really get into it: First, we are interested in your personal experiences, opinions, and thoughts. There are no right and wrong answers, it is all about your personal experiences, views, and perceptions. Feel free to share whatever comes into your mind.

As we described in the information sheet, participation is anonymous. Your statements will be kept completely confidential and thus whatever you say will not have any influence or consequence on you and your employment. The data will be stored and analysed anonymously. It will not be possible to trace any statements back to you as an individual. This is to reassure you that you may also share critique. Please be also reminded that your participation is entirely voluntary. You can refuse to answer specific questions that you do not want to answer and you can stop the interview at any time. Please also let us know if you do not want to answer a specific question.

During the interview, I will be taking notes. This will help me and the research team to better understand the context of the statements, so please do not be confused about this. Also, I would like to ask for your permission to audio-record this interview so that we can stick to your original statements and not misinterpret things that you say. Is this ok for you?

[WAIT FOR PERMISSION AND NOTE IN FIELD NOTES]

What I just explained is also written in the consent form which I would like to ask you to sign.

The interview will last for about 45 minutes.

Do you have any further questions regarding the background of this study or the upcoming interview?

[LEAVE TIME FOR UPCOMING QUESTIONS]
Great! Then let us start

[*Reminder: the leading research questions for this interview are:*

1) ***What made it easy or hard for people to profit from Tosik'aina?***
2) ***What role did it play that Tosik'aina was based on the use of mobile phones and mobile money?***
3) ***What effects that were not planned did Tosik'aina have?***]

[START RECORDING]

Thank you for participating in this interview! Today is the [DATE] and this is the interview with [patient identifier]. I would now like to talk with you about your experiences with visiting a hospital related to the Tosik'aina programme, which took place during the last two years.

Participant identifier: ______________________ Date: ________________

Topic 1	Ice breaker questions
1.1	As said, this interview is about your personal experiences and opinions related to healthcare and financial support for it. This is why I would love to first get to know how you are doing at the moment?
1.2	Let us think back to the last two years, now: How did the pandemic and the famine affect whether you went to see a doctor or other healthcare personnel or not when you needed one, if at all?<br><br>Probes:<br>- Which influences had changes in your community life?<br>- What role did the fear of infection play, if at all?<br>- What role did the government lockdowns play, if at all?<br>- What role did changes in available public transportation options play, if at all?<br>- What role did the costs play, if at all?<br>- How did your use of traditional care vs. CSB/hospital-based healthcare change, if at all?<br>- How did changes in the availability of money influence your use of healthcare, if at all?

I would now like to turn to the Tosik'aina program.

Topic 2	Tosik'aina usage
2.1	When you heard of Tosik'aina, what were your expectations?
2.2	We saw many people who made use of Tosik'aina but also many who went to the hospital but did not use it. I can't talk with all of them, that is why I am here with you and interested in your personal story. Can you tell me your story of how you came to use Tosik'aina?<br><br>Probes:<br>- *Decision-making - process*: You just told me the story of how you came to use Tosik'aina. I would like to understand more about your thoughts at that time. What was in your mind when you had the chance to use Tosik'aina?<br>- *Decision-making - influences:* With whom did you talk about it when you came in contact with Tosik'aina?<br>- *Coverage/usefulness:* We got to know that Tosik'aina was useful for some but not for others. How do you feel about this?<br>- *Accessibility:* [*adapt this probe to the statements made by the participant*] Why was it easy for you to use Tosik'aina?<br>*AND/OR* Why was it (sometimes) difficult for you to use Tosik'aina?

Participant identifier: ________________________ Date: ___________________

2.3	When you went to a health care centre, what about Tosik'aina was different from your expectations, if there is anything?
2.4	How do you understand the payment that had to be made by you as the beneficiary when you went to the hospital and took part in Tosik'aina?<br><br>*[If the interviewee does not know about the co-payment, provide the following definition:]* When you went to the facility and you were registered as a user of Tosik'aina, you had to pay something after you received health care service.<br><br>*[If the participant still cannot speak about his perceptions of the co-payment, you may provide this definition:]* Through Tosik'aina, an NGO paid for 80% of your costs at the centre, you still had to pay 20% of those costs yourself. This part that you paid the payment we are referring to here.<br><br>Probes:<br>- *Perceptions:* What do you think about this co-payment?<br>- *Co-payment knowledge:* What do you know about the height of the co-payment?<br>- *Information sources:* Where did you hear about the details of the co-payment?<br>- -According to you, who "wins" thanks to this system?<br>- - According to you, who "loses" because of this system?<br>- *Way of payment:* How did you make the co-payment? Did that work well for you?
2.5	What were some things that did not work very well for you with Tosik'aina?<br><br>Probes:<br>- *Exclusion of certain populations:* Did you feel some individuals were excluded from making use of the program? If yes: which and why?
2.6	Now, I would like you to think back two years, before Tosik'aina started. Think about a time when you had to see a doctor or nurse. Do you have a concrete situation in mind? (*wait for confirmation of the participant*)<br>When you compare that period to the one that has passed since you learned about Tosik'Aina, what, if anything, has changed in your way of thinking about health care?
2.7	And what did Tosik'aina change in your life, if anything?<br><br>Probes:<br>- *Release of financial burden:* What impact did the financial support have on your life?<br>- *Release of sickness burden:* What impact did receiving treatment for your disease have on your life?<br>- *Social cohesion:* What effect did Tosik'aina have on how you supported each other in the community?

2.8	What were/are people discussing about Tosik'aina?<br><br>Probes:<br>- What did the chef Fokotany tell you about Tosik'aina?<br>- What was discussed in Fivorianas?<br>- *If a woman:* What did you talk about concerning Tosik'aina in the women groups?<br>- Which words come to mind when you think about Tosik'aina in your community?<br>- *Specific rumours:* What were positive and negative things about Tosik'aina that they mentioned?
2.9	With which of the things said do you agree or disagree with?
2.10	If you know of people who have used and/or people who have not been able to use Tosik'Aina, what has Tosik'Aina changed regarding who could go to hospital, if any ?<br><br>Probes:<br>- *Socioeconomic status:* What role did it play how much money people have in whether they were able to use Tosik'aina?

Because Tosik'aina was based on your mobile phone, I would like to ask you some questions about the technology aspect of it.

Topic 3	mTomady usage
3.1	How did you perceive the first introduction to mobile money?<br><br>Probes:<br>- Are you familiar with using mobile money in general ?<br>- Delivery mode: How were you introduced to mobile money?<br>- Was it simple/difficult to understand? To use? If yes/no, why?<br>- What helped you most to understand how to use Tosik'aina?
3.2	In other parts of the world, for example rural Bangladesh, using mobile money could be seen as a challenge for various reasons. One example could be misunderstanding how this technology works or an unreliable mobile network. Did you encounter similar difficulties in using Tosik'aina ?<br><br>Probes:<br>- *Digital literacy:* How familiar were you with mobile money when you had to use it during Tosik'aina?<br>- *Mobile money challenges:* What was challenging about the process of using mobile money, if anything? (i.e. time spent?)

	- *Mobile money dependency:* To what extent do you think you can use mobile money completely independently?<br>- *Infrastructure availability:* What challenges did you face outside of your understanding and confidence with mobile money?<br>*If the participant does not know what to answer, you may probe for the following:* availability of a mobile-money vendor close; Sufficient connection<br>- *Information:* What information would you have needed to cope better with mobile money?<br>- *MM and relative support:* What effect did mobile money have on your ability to receive funds from friends or family members to make your payment?
3.3	What do you think about the use of mobile money to pay for health expenses instead of cash in the future?

Ending the interview

3.4	Now you have provided me with many insights into your experience with Tosik'aina. If you were the designer of Tosik'aina and were to do it again, what are some key things that you would want to change?
	And what would you want to keep ?
3.5	Is there anything I should have asked you but didn't?<br><br>Is there anything else you would like to tell us?
3.6	________<br>*[ask question suggested by participant if applicable]*

Participant identifier: ________________________ Date: ___________________

**Interviewer comments:**
What are your impressions of the interview? Did you observe something special? Was there anything surprising / new for you? Was anything different than in other interviews?
Were there any problems with the interview guide or the related documents?<br>Was the place for the meeting point well chosen ? Is there anything else we could consider changing?

**Reflection**
How do you think this interview went? How difficult was this interview for you?<br>How much do you feel that you affected this interview? What else would be important to add?

Participant identifier: ________________________ Date: ___________________

Participant identifier: _________________________ Date: ____________________

Interview Guide IDIs HCPs

I1	Interview Number	________
I2	Name of Interviewer	____
I3	Interview Date	___ . ___ . ______ (dd.mm.yyyy)
I4	Time Start	___:___
I5	Time End	___:___
I6	Outcome of Interview	☐ Complete<br>☐ Incomplete, reason:<br>___________________________________
I7	Was the interview interrupted? (another person coming in, noises etc.)	☐ No<br>☐ Yes - by whom, how and why?<br>___________________________________

Respondent profile

R1	Interview partner	☐ Head of facility<br>☐ Doctor<br>☐ Nurse<br>☐ Midwife<br>☐ Point of Service<br>☐ Other person: __________________________________
R2	Type of facility	☐ Dispensary

		☐ Reference hospital
R3	Type of facility	☐ Public<br>☐ Private non-faith-based<br>☐ Faith-based
R2	Gender	☐ Male<br>☐ Female<br>☐ Other
R3	Area	☐ Anosy<br>☐ Atsimo-Andrefana<br>☐ Other: ____________________
R4	Age	____ years old

Participant identifier: ______________________ Date: __________________

Thank you very much for agreeing to participate in an interview for our study on Tosik'aina. Our aim in this part of the study is to understand how the program was perceived by the healthcare providers, what worked well, what didn't. Our goal is to improve similar interventions in the future to make them more accessible and beneficial.

I would now like to address a few general things about the project and the interview itself before we really get into it: First, we are interested in your personal experiences, opinions, and thoughts. There are no right and wrong answers, it is all about your personal experiences, views, and perceptions. Feel free to share whatever comes into your mind.

As we described in the information sheet, participation is anonymous. Your statements will be kept completely confidential and thus whatever you say will not have any influence or consequence on you and your employment. The data will be stored and analyzed anonymously. It will not be possible to trace any statements back to you as an individual. This is to reassure you that you may also share critique. Please be also reminded that your participation is entirely voluntary. You can refuse to answer specific questions that you do not want to answer and you can stop the interview at any time. Please also let us know if you do not want to answer a specific question.

During the interview, I will be taking notes. This will help me and the research team to better understand the context of the statements, so please do not be confused about this. Also, I would like to ask for your permission to audio-record this interview so that we can stick to your original statements and not misinterpret things that you say. Is this ok for you?

[WAIT FOR PERMISSION AND NOTE IN FIELD NOTES]

What I just explained is also written in the consent form which I would like to ask you to sign.

The interview will last for about 45 minutes.

Do you have any further questions regarding the background of this study or the upcoming interview?

[LEAVE TIME FOR UPCOMING QUESTIONS]
Great! Then let us start

1) ***What made the implementation of Tosik'aina easier and what made it more difficult?***
2) ***How did Tosik'aina influence whether especially vulnerable populations (i.e. those who have difficulties in using healthcare services) were able to see a doctor or nurse?***
3) ***What effects did Tosik'aina have that were not planned ?***
4) ***What role did it play that Tosik'aina was based on the use of mobile phones and mTomady?***

[START RECORDING]

Thank you for participating in this interview! Today is the [DATE] and this is the interview with [patient identifier] I would now like to talk with you about your experiences, perceptions and views on the Tosik'aina program, including mTOMADY.

Participant identifier: ________________________ Date: ___________________

Topic 1	*Ice breaker questions*
1.1	Before talking about Tosik'aina, I would love to understand your work at your center better. Could you describe what a typical working day looks like for you?
1.2	Could you please explain what your responsibilities were related to the Tosik'aina program in your facility?
1.3	How did the Covid-19 pandemic and the famine affect your work, especially in terms of who came to seek healthcare at your center?<br><br>Probes:<br>- Why did the effect(s) you mentioned occur?<br>- Which group of patients has been especially affected by the pandemic, particularly in their ability to access care in your center?

I would now like to turn to the Tosik'aina program.

Topic 2	*Intervention*
2.1	I will speak with different healthcare providers who took over different roles within Tosik'aina. Today with you, I would like to hear your personal story with Tosik'aina. When you were introduced to Tosik'aina, what were your expectations?<br><br>Probes:<br>- *Way of introduction:* How did the way of introduction to Tosik'aina influence your expectations?<br>- *Compatibility:* What did you think of Tosik'Aina compatibility with existing programs and procedures?<br>- *Worries:* What did you worry about when you first heard about Tosik'aina?
2.2	Throughout the implementation of Tosik'aina, what about Tosik'aina was different from your expectations, if there is anything?<br><br>Probes:<br>- *Presentation vs. reality:* To what extent was the project implemented according to the initial discussions before the implementation?
2.3	What effects did Tosik'aina have at your center?<br><br>Probes:<br>- *Health care provision:* What effects did Tosik'aina have on your ability to provide good healthcare to people who need it?<br>- *Long-term effects:* What long term effects on your center does Tosik'aina have?

	- *Processes:* Which processes did Tosik'aina change? Which did you keep?<br>- *Winners:* Who were the winners of Tosik'aina?<br>- *Losers:* Who were the losers of Tosik'aina?
2.4	What were effects of Tosik'aina that you did not expect?<br><br>*[If participant does not know what to respond, use this]:* We learned from some facilities that Tosik'aina led to an increased workload but this was not the intent of Tosik'aina although the implementers knew about the possibility for it to happen. Do you have other effects like this in mind?<br><br>*[If participant focuses only on negative unexpected effects and you want to probe for positive unintended effects, you may provide this example]:* One facility told us that their quality of care improved because of the claims checks that requested treatment according to the local guidelines but this was not the intent of the study. Do you have other positive effects like this in mind?<br><br>Probes:<br>- How did Tosik'aina affect the availability of personnel and material resources?<br>- What effect did Tosik'aina have on daily life in the Fokotanys?<br>- What effect did Tosik'aina have because it was based on mobile phones and mTomady?<br>- What effect did the claims process, including checking the treatment decisions, have?<br>- What effect did Tosik'aina have on how people viewed healthcare services at the centers?
2.5	Now, let us think through the journey of Tosik'aina, from the moment you got involved in the project until now: What problems did you encounter during this whole time with the implementation of Tosik'aina?<br><br>Probes:<br>- *Communication within NGO:* Which problems arose regarding the communication within Doctors for Madagascar?<br>- *Monthly thresholds:* What do you think about the maximal amount of claims costs that DFM would cover for your center?<br>- Claim thresholds: What do you think about the maximal<br>- *Payments:* What role did payment procedures play?<br>- *Digitization:* What role played the fact that claims were sent through mTomady and payments were made through mobile money?
2.6	What do you think about the copayment that patients had to make?

	[If the interviewee does not know about the co-payment, provide the following definition:] When an individual eligible for Tosik'aina visited your facility and was registered as a Tosik'aina user, he had to pay part of the costs after he had received health care service.<br><br>Probes:<br>- *Amount of patient contribution:* What do you think of the height of the patient contribution?<br>- *Communication:* How did the communication of this co-payment work?<br>- *For CMDT:* What differences did you see for the group of malaria patients that did not have to make any contributions?
2.7	How did Tosik'aina change the trust patients have in the health services you provide, if at all?<br><br>Probes:<br>- *Center perception:* How did Tosik'aina change how patients view your services, if at all?
2.8	What did/do you hear people say about Tosik'aina?<br><br>Probes:<br>- *Specific groups:*<br>- from other health care providers<br>- In the communities<br>- Own staff/colleagues<br>- *Effects:* Did those affect your work?
2.9	Which patient groups did you identify that would have needed Tosik'aina most but were not able to access it for different reasons?<br><br>Probes:<br>- *Mechanisms:* Why was it difficult for the people you mentioned to access Tosik'aina?<br>- *Reason in design:* What influence did the design of Tosik'aina have on not allowing the inclusion of these people?<br>- *Distance to facility:* What role did distance to facility play?<br>- *Sensitization:* What role did not reaching individuals through sensitization campaigns play?<br>- *Eligibility:* Which groups were excluded because of the eligibility criteria?<br>- *Eligibility effects:* What tensions did the fact create that some patients were eligible for Tosik'aina and others not, if at all?<br>- *Digital literacy:* What role did familiarity with mobile money play?
2.10	What could have been done to include these groups?

Participant identifier: ________________________ Date: ___________________

I would now like to turn to the digital tool called "mTomady" that we used in the Tosik'aina program. Is mTomady the term you would use as well or shall we refer to it with a different word?

Topic 3	*Digitization*
3.1	How did you perceive the first introduction to mTomady?<br><br>Probes:<br>- Was it simple/difficult to understand? To use?
3.2	How did mTomady impact your workload and/or your workflow, if at all?<br><br>Probes:<br>- *If increased workload:* Why did it increase your workload?<br>- *If decreased workload:* Why did it decrease your workload?<br>- How did it change your workflow?
3.3	What do you think of the use of mTomady in your working context?<br><br>Probes:<br>- What are the biggest advantages brought to your work by mTomady?<br>- What are the biggest inconveniences brought to your work by mTomady?<br>- What could be improved in mTomady to make it more effective?
3.4	How appropriate is technology in the context you work in?<br><br>Probes:<br>- To what extent does it align with<br>- Availability of electricity<br>- Digital literacy in the population<br>- Availability of network connection<br>- Mobile phone access

Ending the interview

4.1	If you were the designer of Tosik'aina with all the knowledge you have now, what would be some key things that you would change about the intervention?<br><br>Probes<br>- *Reasons*: Why do you think so?
4.2	And what are key elements of the intervention that you would want to keep?<br>- *Reasons*: Why do you think so?

Participant identifier: ________________________ Date: ___________________

4.3	Is there anything I should have asked you but didn't?<br><br>Is there anything else you would like to tell us?
4.4	________<br>*[ask question suggested by participant if applicable]*

Participant identifier: ________________________ Date: ___________________

Interviewer comments

**Interviewer comments:**
What are your impressions of the interview? Did you observe something special? Was there anything surprising / new for you? Was anything different than in other interviews?
Were there any problems with the interview guide or the related documents?<br>Was the place for the meeting point chosen well? Is there anything else we could consider changing?

Participant identifier: ________________________ Date: ___________________

**Reflection**
How do you think this interview went? How difficult was this interview for you?<br>How much do you feel that you affected this interview? What else would be important to add?

Participant identifier: ______________________ Date: __________________

Participant identifier: ________________________ Date: ___________________

Interview Guide IDIs Policy Makers

I1	Interview Number	_______
I2	Name of Interviewer	___________________________________
I3	Interview Date	___ . ___ . ______ (dd.mm.yyyy)
I4	Time Start	___:___
I5	Time End	___:___
I6	Outcome of Interview	☐ Complete<br>☐ Incomplete, reason:<br>_______________________________
I7	Was the interview interrupted? (another person coming in, noises etc.)	☐ No<br>☐ Yes - by whom, how and why?<br>_______________________________

Respondent profile

R1	Employment level	☐ Médecin Inspecteur<br>☐ Directeur régional de santé (DRS)
R2	Gender	☐ Male<br>☐ Female<br>☐ Other

Participant identifier: _________________________ Date: ____________________

R3	Area	☐ Anosy<br>☐ Atsimo-Andrefana<br>☐ Other: ____________________
R4	Age	_____ years old

Participant identifier: _______________________ Date: __________________

Thank you very much for agreeing to participate in an interview for our study on Tosik'aina. Our aim in this part of the study is to understand how the program was perceived by policy makers, what worked well, what didn't. Our goal is to improve similar interventions in the future to make them more accessible and beneficial.

I would now like to address a few general things about the project and the interview itself before we really get into it: First, we are interested in your personal experiences, opinions, and thoughts. There are no right and wrong answers, it is all about your personal experiences, views, and perceptions. Feel free to share whatever comes into your mind.

As we described in the information sheet, participation is anonymous. Your statements will be kept completely confidential and thus whatever you say will not have any influence or consequence on you and your employment. The data will be stored and analyzed anonymously. It will not be possible to trace any statements back to you as an individual. This is to reassure you that you may also share critique. Please be also reminded that your participation is entirely voluntary. You can refuse to answer specific questions that you do not want to answer and you can stop the interview at any time. Please also let us know if you do not want to answer a specific question.

During the interview, I will be taking notes. This will help me and the research team to better understand the context of the statements, so please do not be confused about this. Also, I would like to ask for your permission to audio-record this interview so that we can stick to your original statements and not misinterpret things that you say. Is this ok for you?

[WAIT FOR PERMISSION AND NOTE IN FIELD NOTES]

What I just explained is also written in the consent form which I would like to ask you to sign.

The interview will last for about 45 minutes. Do you have any further questions regarding the background of this study or the upcoming interview?

[LEAVE TIME FOR UPCOMING QUESTIONS]
Great! Then let us start

[*Reminder: the leading research questions for this interview are:*

1) ***What made the implementation of Tosik'aina easier and what made it more difficult?***
2) ***How did Tosik'aina influence whether especially vulnerable populations (i.e. those who have difficulties in using healthcare services) were able to see a doctor or nurse?***
3) ***What effects did Tosik'aina have that were not planned ?***
4) ***What role did it play that Tosik'aina was based on the use of mobile phones and mTomady?***]

[START RECORDING]

Participant identifier: ________________________ Date: ___________________

Thank you for participating in this interview! Today is the [DATE] and this is the interview with [patient identifier] I would now like to talk with you about your experiences, perceptions and views on the Tosik'aina program, including mTOMADY.

For this study we will be talking to several people, including beneficiaries of the program and health care providers to learn about their perspectives on the program. From your position as a policy maker and someone with an overview of the whole district/region, we hope to understand how this project has affected health care not only in singular institutions but throughout the region.

Now, I would like to turn to the Tosik'aina program.

Topic 1	*Tosik'aina*
1.1	I would like to directly take you into the intervention of Tosik'aina. Let us think through the journey of Tosik'aina, from the moment you first heard about the project until now: What worked well in your area during this whole time with the implementation of Tosik'aina?
1.2	Now, let us think through the journey of Tosik'aina, from the moment you first heard about the project until now: What problems arose in your area during this whole time with the implementation of Tosik'aina?<br><br>Probes:<br>- *Communication with the NGO:* Which problems arose regarding the communication with Doctors for Madagascar?
1.3	What effects of Tosik'Aina on collaborating/participating centers have you seen, if any?<br><br>Probes:<br>- *Financial effects - users:* What effect has Tosik'Aina had on the collaborating centers' financial situation, if any?<br>- *Negative effects:* Which negative effects did the intervention have? [*If the participant does not know what to respond or does not give much detail, probe for the following aspects*]<br>- Limited personnel and material resources<br>- Cultural inappropriateness<br>- Technology<br>- Claims process<br>- Lack of trust in project implementers<br>- Negative effects on other facilities (e.g. lack of patients and thus lack of income)<br>- Dependency on external money and skills
1.4	What effects of Tosik'aina on centers without Tosik'aina did you see, if any?

	Probes:<br>- *Comparison:* What differences do you see between centers using<br>- Tosik'Aina and centers that have not?<br>- *Financial effects - non-users:* What effect did Tosik'Aina have on non-collaborating centers' financial situation, if any?
1.5	How could Tosik'aina be adapted to align better with the health priorities on your agenda?
1.6	*[present the sheet with preliminary results from our quantitative evaluation]* These are preliminary findings from our data on the effect of Tosik'aina on health care utilization . What do you think about this data?
1.7	What were the effects of Tosik'Aina that you did not expect?<br><br>*[If participant does not know what to respond, use this]:* We learned from some facilities that Tosik'aina led to an increased workload but this was not the intent of Tosik'aina although the implementers knew about the possibility for it to happen. Do you have other effects like this in mind?<br><br>*[If participant focuses only on negative unexpected effects and you want to probe for positive unintended effects, you may provide this example]:* One facility told us that their quality of care improved because of the claims checks that requested treatment according to the local guidelines but this was not the intent of the study. Do you have other positive effects like this in mind?<br><br>Probes:<br>- How did Tosik'aina affect the availability of personnel and material resources?<br>- What effect did Tosik'aina have on daily life in the Fokotanys?<br>- What effect did Tosik'aina have because it was based on mobile phones and mTomady?<br>- What effect did the claims process, including checking the treatment decisions, have?<br>- What effect did Tosik'aina have on how people viewed healthcare services at the centers?
1.8	Which patient groups did you identify that would have needed Tosik'aina most but were not able to access it for different reasons?<br><br>Probes:<br>- *Mechanisms:* Why was it difficult for the people you mentioned to access Tosik'aina?<br>- *Reason in design:* What influence did the design of Tosik'aina have on not allowing the inclusion of these people?<br>- *Distance to facility:* What role did distance to facility play?

	- *Sensitization:* What role did not reaching individuals through sensitization campaigns play?<br>- *Eligibility:* Which groups were excluded because of the eligibility criteria?<br>- *Digital literacy:* What role did familiarity with mobile money play?
1.9	What could have been done to include these groups?
1.10	When you were introduced to Tosik'aina the first time, what were your expectations?<br><br>Probes:<br>- *Way of introduction:* How did the way of introduction to Tosik'aina influence your expectations?<br>- *Compatibility:* What did you think of Tosik'Aina's compatibility with existing programs and procedures?<br>- *Worries:* What did you worry about when you first heard about Tosik'aina?
1.11	Throughout the planning and implementation of Tosik'aina, what was different from your expectations, if there is anything?<br><br>Probes:<br>- *Presentation vs. reality:* To what extent was the project implemented according to the initial discussions before the implementation?

Overall perspective and ending

2.1	If you were the designer of Tosik'aina with all the knowledge you have now, what would be some key things that you would change about the intervention?<br><br>Probes<br>- What would you do if you had the same budget available?<br>- What would you do if you had an unlimited budget?<br>- *Reasons*: Why do you think so?
2.2	And what are key elements of the intervention that you would want to keep?<br>- *Reasons*: Why do you think so?
2.3	Is there anything I should have asked you but didn't?<br><br>Is there anything else you would like to tell us?
2.4	________<br>*[ask question suggested by participant if applicable]*

Participant identifier: ________________________ Date: ___________________

Participant identifier: ________________________ Date: ___________________

Interviewer comments (all interviews)

**Interviewer comments:**
What are your impressions of the interview? Did you observe something special? Was there anything surprising / new for you? Was anything different than in other interviews?
Were there any problems with the interview guide or the related documents?<br>Was the place for the meeting point chosen well? Is there anything else we could consider changing?

Participant identifier: ________________________ Date: ___________________

**Reflection**
How do you think this interview went? How difficult was this interview for you?<br>How much do you feel that you affected this interview? What else would be important to add?

Participant identifier: ________________________ Date: ___________________

Interview Guide IDIs Project Staff

I1	Interview Number	_______
I2	Name of Interviewer	___________________________________
I3	Interview Date	___ . ___ . ______ (dd.mm.yyyy)
I4	Time Start	___:___
I5	Time End	___:___
I6	Outcome of Interview	☐ Complete<br>☐ Incomplete, reason:<br>_______________________________
I7	Was the interview interrupted? (another person coming in, noises etc.)	☐ No<br>☐ Yes - by whom, how and why?<br>_______________________________

Respondent profile

R1	Interview partner	☐ Claims team<br>☐ Finance team<br>☐ Implementation team<br>☐ Other person: ______________________________
R2	Gender	☐ Male<br>☐ Female

Participant identifier: ______________________________ Date: ________________________

		☐ Other
R3	Area	☐ Antananarivo<br>☐ Anosy<br>☐ Atsimo-Andrefana<br>☐ Other: ______________________
R4	Age	_____ years old

Participant identifier: _______________________ Date: __________________

Thank you very much for agreeing to participate in an interview for our study on Tosik'Aina. Our aim in this part of the study is to understand how the program was perceived by the project staff, what worked well, what didn't. Our goal is to improve similar interventions in the future to make them more accessible and beneficial.

I would now like to address a few general things about the project and the interview itself before we really get into it: First, we are interested in your personal experiences, opinions, and thoughts. There are no right and wrong answers, it is all about your personal experiences, views, and perceptions. Feel free to share whatever comes into your mind.

As we described in the information sheet, participation is anonymous. Your statements will be kept completely confidential and thus whatever you say will not have any influence or consequence on you and your employment. The data will be stored and analysed anonymously. It will not be possible to trace any statements back to you as an individual. This is to reassure you that you may also share critique. Please be also reminded that your participation is entirely voluntary. You can refuse to answer specific questions that you do not want to answer and you can stop the interview at any time. Please also let us know if you do not want to answer a specific question.

During the interview, I will be taking notes. This will help me and the research team to better understand the context of the statements, so please do not be confused about this. Also, I would like to ask for your permission to audio-record this interview so that we can stick to your original statements and not misinterpret things that you say. Is this ok for you?

[WAIT FOR PERMISSION AND NOTE IN FIELD NOTES]

What I just explained is also written in the consent form which I would like to ask you to sign.

The interview will last for about 45 minutes.

Do you have any further questions regarding the background of this study or the upcoming interview?

[LEAVE TIME FOR UPCOMING QUESTIONS]
Great! Then let us start

[START RECORDING]

[*Reminder: the leading research questions for this interview are:*

1) ***What made the implementation of Tosik'aina easier and what made it more difficult?***
2) ***How did Tosik'aina influence whether especially those who have difficulties in using healthcare services were able to see a doctor or nurse?***
3) ***What effects did Tosik'aina have that were not planned ?***
4) ***What role did it play that Tosik'aina was based on the use of mobile phones and mTomady?***]

Thank you for participating in this interview! Today is the [DATE] and this is the interview with [patient identifier] I would now like to talk with you about your experiences, perceptions and views on the Tosik'aina program, including mTOMADY.

Participant identifier: ________________________ Date: ___________________

Topic 1	*Ice breaker questions*
1.1	Could you describe what a typical working day with DFM looks like for you, and during Tosik'aina in particular?

Topic 2	*Effectiveness*
2.1	I will speak with people who had different roles in Tosik'aina. Today with you, I would like to hear your personal story with Tosik'aina. When you started working on Tosik'aina, what were your expectations?<br><br>Probes:<br>- *Compatibility:* What were your thoughts on how well Tosik'aina would be compatible with existing programmes and procedures?<br>- *Worries:* What did you worry about when you first heard about Tosik'aina?
2.2	How did your perceptions of Tosik'aina change over the course of the implementation?
2.3	How was the first introduction to Tosik'Aina?<br><br>Probes:<br><br>- Understanding Technology: How easy or difficult was it for you and your team to understand the technological aspects of Tosik'aina (mTomady, mobile money)? Why/why not?<br>- Using Technology: How easy or difficult was it for you to use the mTomady platform during the implementation of Tosik'aina? Why?
2.4	Now, let us think through the journey of Tosik'aina, from the moment you got involved in the project until now: What problems did you face during the implementation of Tosik'aina?<br><br>Probes:<br>- *Technology:* What problems did you face with mTomady as a digital platform?<br>- *Communication with provider staff:* Which problems arose regarding the communication with health care facility staff?<br>- *Internal communication within the NGO:* Which problems arose regarding the communication within Doctors for Madagascar and<br>- *Communication with a digital provider:* Which problems arose regarding the communication with mTomady?

Participant identifier: ________________________ Date: ___________________

	- *Problems at specific points of time*: [*Provide participant with certain steps of the implementation to hear about the challenges they faced*]<br>- Onboarding of new facilities (contracts, set-up, agent, etc.)<br>- Patient registration and validation<br>- Claim filing<br>- Claim review (including those exceeding maximum amount, claims with incomplete data)<br>- Invoice generation and payment<br>- Which problems arose regarding the structure of established processes?
2.5	What could be or has been done to solve these challenges?
2.6	What helped to implement successfully Tosik'aina?<br><br>Probes:<br>- *Key enablers:* What are the essential aspects without which the intervention would not have worked?<br>- *Technology:* How did technology help during the implementation?<br>- *Enablers in initiation phase:* What was helpful for the project in the set-up at and onboarding of new facilities?<br>○ Contract negotiation<br>○ Training<br>○ Sensitization<br>- *Enablers in routine phase:* What was helpful for the project after the onboarding of the centers was completed?<br>- *Communication with provider staff:* What was helpful regarding the communication with health care facility staff?<br>- *Communication within the organization:* What was helpful regarding the communication within Doctors for Madagascar?<br>- *Communication within the digital provider:* What was helpful regarding the communication with mTomady?<br>- *Process structures (claims, payment):* What was helpful regarding the structure of established processes?
2.7	What were the effects of Tosik'aina that you did not expect?<br><br>*[If participant does not know what to respond, use this]: As a positive unexpected effect* one facility told us that their quality of care improved because of the claims checks that requested treatment according to the local guidelines. As a negative unexpected effect we heard about added bureaucracy and workload for the health care providers. Do you have other cases like this in mind?<br><br>Probes:<br>- How did Tosik'aina affect your/DFM's relationship with the centers?

	- How did Tosik'aina affect the availability of personnel and material resources?<br>- What effect did Tosik'aina have on daily life in the Fokotanys?<br>- What effect did Tosik'aina have because it was based on mobile phones and mTomady?<br>- What effect did the claims process, including checking the treatment decisions, have?<br>- What effect did Tosik'aina have on how people viewed healthcare services at the centers?
2.8	Tosik'aina was intended to help especially very vulnerable groups. What do you think about how this goal was reflected in the design and implementation of Tosik'aina?<br><br>Probes:<br>- How did the Tosik'aina project team try to include especially vulnerable groups during the implementation phase?<br>- Which vulnerable groups were considered in particular?<br>- indigenous groups<br>- certain age-related groups<br>- disabled populations<br>- certain language barriers<br>- low-income individuals<br>- Remote populations with limitations regarding internet access<br>- Female gender<br>- stigmatized conditions (HIV/TB/…)<br>- How were the needs and characteristics of such groups taken into account?
2.9	With the knowledge you have now after the end of Tosik'aina, which patient groups did you identify that would have needed Tosik'aina most but were not able to access it for different reasons, if there are any?<br><br>Probes:<br>- *Mechanisms:* Why was it difficult for the people you mentioned to access Tosik'aina?<br>- *Reason in design:* What influence did the design of Tosik'aina have on not allowing the inclusion of these people?<br>- *Distance to facility:* What role did distance to facility play?<br>- *Sensitization:* What role did not reaching individuals through sensitization campaigns play?<br>- *Eligibility:* Which groups were excluded because of the eligibility criteria?<br>- *Digital literacy:* What role did familiarity with mobile money play?
2.10	What could have been done to include these groups?

Participant identifier: ________________________ Date: ___________________

Topic 2	*Technology*
3.1	What do you think about the use of mTomady for Tosik'aina?<br><br>Probes:<br>- What are the biggest advantages brought to your work by mTomady?<br>- What are the biggest inconveniences brought to your work by mTomady?<br>- What could be improved in mTomady to make it more effective?
3.2	How appropriate is technology in the context you work in?<br><br>Probes:<br>- To what extent does it align with<br>- Availability of electricity<br>- Digital literacy in the population<br>- Availability of network connection<br>- Mobile phone access

Ending the interview

4.1	If you were the designer of Tosik'aina with all the knowledge you have now and were to do it again, what would be some key things that you would change about the intervention?<br><br>Probes<br>- *Reasons*: Why do you think so?
4.2	And what are key elements of the intervention that you would want to keep?<br>- *Reasons*: Why do you think so?
4.3	Is there anything I should have asked you but didn't?<br><br>Is there anything else you would like to tell us?
4.4	________<br>*[ask question suggested by participant if applicable]*

Participant identifier: ________________________ Date: ___________________

Interviewer comments

**Interviewer comments:**
What are your impressions of the interview? Did you observe something special? Was there anything surprising / new for you? Was anything different than in other interviews?
Were there any problems with the interview guide or the related documents?<br>Was the place for the meeting point chosen well? Is there anything else we could consider changing?

Participant identifier: ________________________ Date: ___________________

**Reflection**
How do you think this interview went? How difficult was this interview for you? How much do you feel that you affected this interview? What else would be important to add?
